# Healthcare professionals’ perspectives on the barriers and facilitators of integrated childhood obesity care

**DOI:** 10.1186/s12913-024-11532-9

**Published:** 2024-09-27

**Authors:** Emma van den Eynde, Bibian van der Voorn, Leandra Koetsier, Hein Raat, Jaap C. Seidell, Jutka Halberstadt, Erica L. T. van den Akker

**Affiliations:** 1 Sophia Children’s Hospital, Department of Pediatrics, Division of Pediatric Endocrinology and Obesity Center CGG, Erasmus MC University Medical Center, Rotterdam, The Netherlands; 2 Department of Health Sciences, Faculty of Science, Vrije Universiteit Amsterdam, Amsterdam Public Health Institute, Amsterdam, The Netherlands; 3 Department of Public Health, Erasmus MC University Medical Center, Rotterdam, The Netherlands

**Keywords:** Pediatric obesity, Integrated care, Patient-centered Care, Complexity, Healthcare providers, Qualitative research

## Abstract

**Background:**

Both the causes and consequences of childhood obesity can be complex. To provide healthcare that is suitably tailored to the specific needs of children with obesity integrated care is required. The objective of this study was to explore the perceived barriers and facilitators of healthcare professionals (HCPs) in providing integrated care for children with obesity, to support them in tailoring the healthcare approach.

**Methods:**

In this qualitative study, semi-structured in-depth interviews were conducted with 18 healthcare professionals with experience in childhood obesity care; pediatricians, youth healthcare nurses and a youth healthcare physician. A two-phased thematic content analysis was performed: an inductive analysis with open and selective coding and a deductive analysis with axial coding using the patient-centered care model by Stewart.

**Results:**

Overall, the healthcare professionals defined the etiology of obesity as complex, and experienced the integrated care as complicated. The results fit into the four theme-structure of the patient-centered care model, with the integrated care system as an additional fifth theme. The main barriers were perceived within the sub-themes of illness and healthcare experiences, and sensitivity over talking about weight-related issues. The main facilitators were perceived within the sub-themes of conducting a biomedical, psychosocial and lifestyle assessment, tailoring the approach to families’ situation and investing in a family-professional relationship. Weight stigma appeared to be an underlying barrier for healthcare professionals that impacted, both explicitly and implicitly, upon all themes.

**Conclusions:**

Healthcare professionals providing integrated care for children with obesity, experience this type of care as complicated and comprising many barriers and facilitators regarding the four themes of the patient-centered care model and the fifth theme of the integrated care system. This paper demonstrates the patient-centered care model could prove helpful structuring a tailored approach within integrated care. This approach supports healthcare professionals in adopting a broad perspective towards individual and environmental factors and investing in the relationship, with respect to the sensitivity and complexity of childhood obesity.

**Supplementary Information:**

The online version contains supplementary material available at 10.1186/s12913-024-11532-9.

## Background

The number of children with obesity has increased rapidly worldwide over the past four decades [1]. In 2020, 14.7% of children in the Netherlands aged 4 to 17 were overweight, of whom 2.5% had obesity [2]. The etiology and maintenance of childhood obesity is shaped by a complex interaction between many factors across different levels. At the individual level, these can be biological and psychosocial factors, while at the environmental level, culture and weight stigma as a moderating factor for pyschosocial health can play an important role [3, 4].

Obesity can have negative short- and long-term physical and psychosocial consequences [5–7]. Effective treatment requires integrated care to achieve and maintain behavioral change towards a healthier lifestyle [8–10]. The recently developed Dutch ‘National model integrated care for childhood overweight and obesity’ describes how effective obesity support and care goes beyond a mere healthy lifestyle, and instead also necessitates paying attention to the underlying individual and environmental causes [8, 11]. This model requires professionals from within the healthcare and social domain to work dynamically together, with the support of a coordinating professional, who identifies and monitors children with obesity, manages their care, performs diagnostics for underlying causes when required, and organizes interdisciplinary collaborations [12]. The basis for the Dutch ‘National model integrated care’ was the chronic care model of Wagner which is a form of patient-centered care [11, 13–15]. While there are many definitions of patient-centered care, these generally include the following characteristics: sharing power and responsibility, therapeutic alliance, patient-as-person, biopsychosocial perspective, doctor-as-person and coordinated care [16–18]. These characteristics can be traits of either the healthcare system itself or individual healthcare professionals (HCPs) [13]. One predominant clinical method for carrying out patient-centered care was put forward by M Stewart, JB Brown, WW Weston, IR McWhinney, CL McWilliam and T Freeman [19], who operationalized it as comprising four interactive components: (1) Exploring health, disease, and the illness experience, (2) understanding the whole person and its contexts, (3) finding common ground and (4) enhancing the patient-professional relationship. When it comes to children with (or at risk for) chronic diseases, a coordinating professional can have a designated central role within patient-centered care.

The barriers and facilitators in healthcare for children with obesity that have been identified in prior research relate to both the levels of the individual child, parent or professional, that is, a lack of problem awareness or self-efficacy, and the broader social and physical environment, for example, the unsupportive organization of healthcare or the obesogenic environment [20]. Facilitators consist of respectful supportive relations with the social environment, including with HCPs [21, 22]. To minimize the burden of these barriers, it is recommended that childhood obesity care addresses these factors, alongside the needs and possibilities of children and parents themselves [11, 19, 23–26]. Although the patient-centered care model comprises such factors, relatively little research has been done exploring the perspectives of HCPs providing this form of integrated care. It is important to gain insight into their perspectives, with this study aiming to support them in tailoring the healthcare approach. Consequently, the research question underpinning this study is: what do healthcare professionals perceive as the barriers and facilitators to providing integrated care in childhood obesity?

## Methods

A qualitative study design consisting of in-depth semi-structured interviews was adopted in order to study the two research questions regarding healthcare professionals’ (HCPs’) perspective on barriers and facilitators for (1) children with obesity and their parents in achieving a healthier lifestyle and (2) the integrated care for those children and parents, which is described in this paper. The results of the first research question are published elsewhere [27]. The same methods were used for both papers, and the COREQ checklist was used to increase the level of rigor in both the carrying out of the study and its subsequent description [28].

### Participants and recruitment

To include various perspectives, HCPs from varying professions and with different responsibilities when providing integrated care, were recruited to participate. These included pediatricians, youth healthcare nurses and youth healthcare physicians. Professionals working in the Dutch youth healthcare (YHC) system perform regular medical check-ups of all children from 0–19 years old [29]. The following HCPs were eligible for inclusion: (1) pediatricians from centers of expertise for childhood obesity, as defined by the Dutch association for pediatrics, (2) YHC nurses and physicians from a municipality that contributed to the development of the ‘National model integrated care for childhood overweight and obesity’ and (3) YHC nurses who have experience with the role of coordinating professional as part of local integrated care. Participants were recruited across the Netherlands, within both smaller and larger municipalities.

### Data collection

Eighteen semi-structured interviews were conducted, each of which lasted approximately 60 min. An interview guide was used during the interviews (see Supplement A with the interview protocol, for more information on informed consent, how the interview was introduced to the participant and the questions). The interviews were audio-recorded, transcribed verbatim and anonymized. The interviewer kept field notes with reflections on the interviews. A member check was done with a summary of the interview [30]. While many sub-themes were reported, as the study had a very broad focus, reaching saturation on all sub-themes was not possible.

### Data analysis

A thematic content analysis was performed using the program MAXQDA 2018 [31]. To ensure triangulation of the researchers, the data was coded and analyzed by two researchers (EvdE and LK). The analysis was an iterative process and changes were logged. The researchers (EvdE and LK) first familiarized themselves with the data by reading over the transcripts and summarizing them. The subsequent analysis comprised two phases. In the first inductive phase, open coding was done independently by the two researchers. Two coded transcripts were compared to achieve consensus on a preliminary set of codes. Subsequently, both researchers independently created a coding tree and then compared them to establish consensus over a preliminary coding tree. The remaining part of the transcripts were then axially coded by the main researcher (EvdE) into sub-themes using this coding tree. In the second deductive phase, with selective coding, the four components of the patient-centered model (Stewart et al., 2013) were used as themes to structure the sub-themes. The findings were discussed with all co-authors and a consensus was established.

## Results

Twenty healthcare professionals (HCPs) were approached to participate in the study, of which one did not respond, and one did not have time to take part in an interview. Resultantly, 18 HCPs (17 females and one male) with a mean age of 43.8 years old (range 29 – 60 years) participated: six pediatricians, 11 YHC nurses and one YHC physician. They had an average working experience of 8.7 years (range 1 – 18 years, 5 unknown) with children with obesity and had worked in eight different municipalities across the Netherlands. All participating HCPs worked with the ‘National model integrated care for childhood overweight and obesity’. Eight of them worked with children aged 4–12 years old, one HCP worked with children aged 12–18 years old, while nine HCPs worked with children aged 0 to 18 years old.

Words very frequently mentioned were ‘complicated’ and ‘complex’: 470 times in total in around 18 h of interviews, which amounts to once every two-and-a-half minute on average. Different aspects of integrated childhood obesity care were labeled in this way, namely interrelating factors that influence both the etiology and maintenance of obesity, the necessity of close collaboration with a network of HCPs providing integrated care, and their general experience regarding the complicated nature of providing this type of care.

**Fig. 1:**
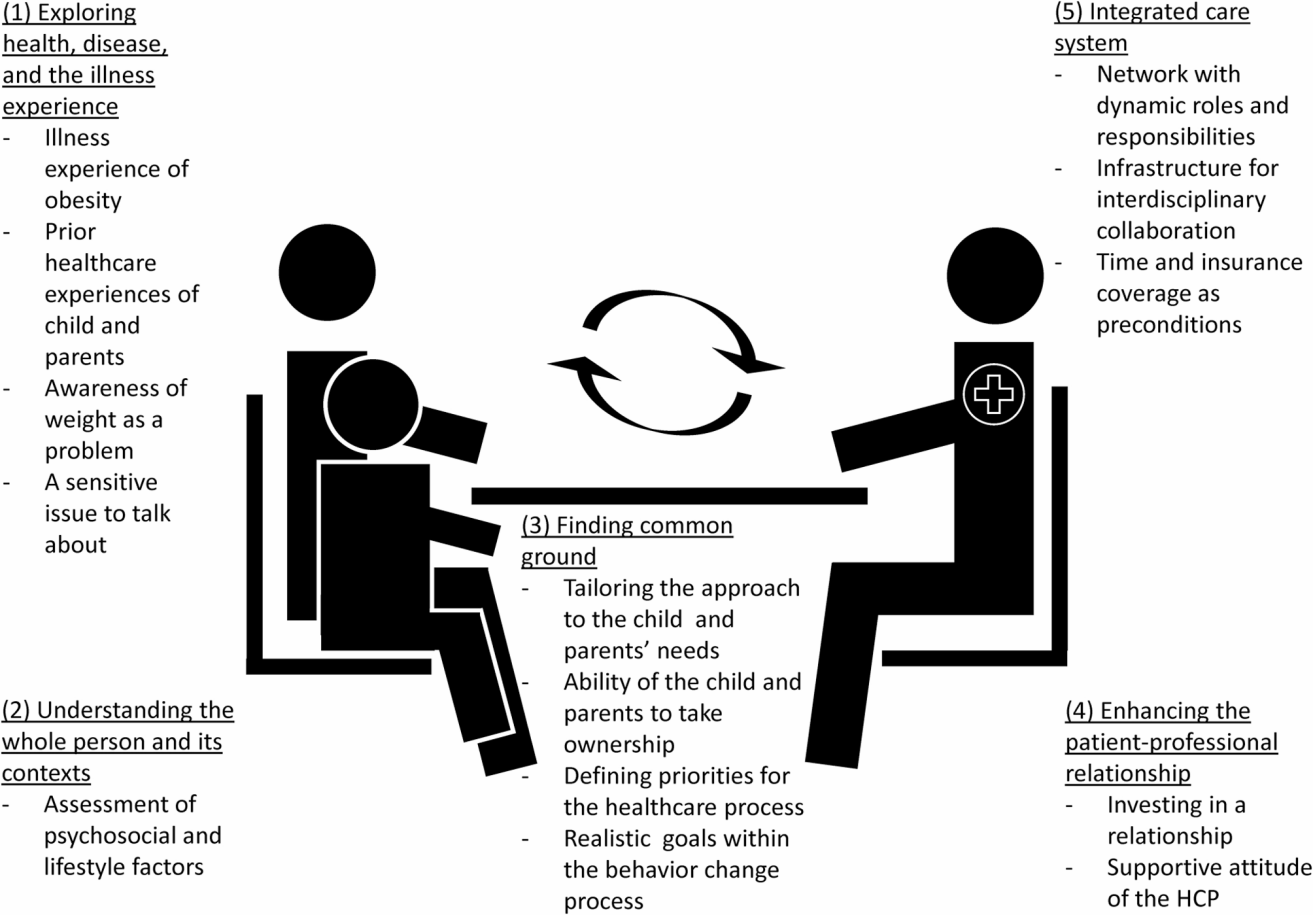
Identified themes and-subthemes structured by the patient-centered care model within integrated care for childhood obesity, adjusted from M Stewart, JB Brown, WW Weston, IR McWhinney, CL McWilliam and T Freeman [19]

The HCPs perceived many barriers and facilitators within all four themes of the patient-centered care model by M Stewart, JB Brown, WW Weston, IR McWhinney, CL McWilliam and T Freeman [19] (Fig. 1, themes 1–4), ranging from general and theoretical to more practical. In addition, the HCPs also perceived barriers and facilitators related to the organization of healthcare, which were bundled within the fifth theme ‘Integrated care system’ (Fig. 1, theme 5). The 14 sub-themes emerging out of our analysis of the interviews are described in turn below (see also Fig. 1 and Tables 1, 2, 3, 4 and 5).

**Table 1:** HCPs’ perceived barriers and facilitators related to the theme ‘Exploring health, disease, and the illness experience’

**Sub-themes**	*Barriers*	*Facilitators*
**Illness experience of obesity**	Child reports not being able to keep up with physical activities	*See facilitators in sub-theme ‘Supportive attitude of the HCP’*
	Child reports mental health problems related to obesity and feelings of shame or low self-confidence	
	Child reports being bullied	
	Child and parents report feeling judged by society	
**Prior healthcare experiences of child and parents**	Child and parents report experiences of failure in behavior change, while often not received appropriate lifestyle intervention	*See facilitators in sub-theme 'Investing in a relationship’*
	Child and parents report having both a long treatment history and seeing a lot of different professionals	
	Child and parents report that they have told their story too many times	
	Child and parents report that they received either overly difficult or insufficiently practical advice	
	Child reports feeling undervalued and misunderstood by HCPs	
**Awareness of weight as a problem**	At YHC, families seem less aware of the problem, perhaps because they do not initiate the appointment themselves	Problem awareness tends to be higher amongst older children because of comparison to peer-group
	Child and parents are unaware of the obesity or its health consequences	HCP assesses stage of change, to tailor their approach
		Experiencing the burden of obesity
**A sensitive issue to talk about**	Parents seem to feel personally attacked when HCP address their child’s weight	Finding a balance between not focusing too much on the weight itself, but still addressing it
	Talking about weight appears to place pressure upon the child and parents	Making weighing optional
	Using growth charts can be confronting and might negatively impact on the relationship	Using growth charts can help as an objective tool
		Being sensitive in choosing appropriate words

**Table 2:** HCPs’ perceived barriers and facilitators related to the theme ‘Understanding the whole person and its contexts’

**Sub-themes**	*Barriers*	*Facilitators*
**Assessment of psychosocial and lifestyle factors**	Complexity of factors involved in the development and maintenance of obesity	Gathering information about the broader situation, to tailor the healthcare process
	Assessment and treatment of solely nutrition and physical activity	Involvement of the whole family, including father and grandparents
	Complexity of multi-problem families	Mapping other involved professionals
		Basic knowledge of the complexity of factors influencing the etiology and maintenance of obesity

**Table 3:** HCPs’ perceived barriers and facilitators related to the theme ‘Finding common ground’

**Sub-themes**	*Barriers*	*Facilitators*
**Tailoring the approach to the child and parents’ needs**	HCP has too little knowledge of non-Dutch cultural practices and lacks the facilities to overcome the language barrier	Tailoring the approach to characteristics of the child and parents, by adjusting logistical aspects as well as the type of support and care
	Organisational issues sometimes hinder HCP’s ability to tailor their approach	Experimenting with different strategies by using creativity and flexibility
	Giving advice before knowing the situation and establishing a relationship	Sitting on your hands, letting the child and parents set the pace of the process
	HCP finds it difficult to have patience and invest in a relationship, while not directly focusing on a solution	Using conversational techniques (probing, listening, paying attention, silence) and methods (solution-focused therapy or motivational interviewing)
		Having physical materials, to support and illustrate questions or information
**Ability of the child and parents to take ownership**	HCP reports that children or parents are not always able or willing to take ownership	Creating ownership of the behavior change process and the action plan; Let child and parents determine what to (not) talk about
	HCP has obligations in the healthcare process, due to protocols or registration tasks	HCPs adopting a coaching role, by combining children, parents and HCPs’ knowledge, skills, and possibilities
**Defining priorities for the healthcare process**		Defining a family’s priorities and requests, related to the obesity and their life generally
		Addressing barriers and needs within the behavior change and healthcare process
		Sometimes it is necessary to prioritize psychosocial factors, prior to starting the process of lifestyle change
**Realistic goals within the behavior change process**	HCP reports that child, parents, or HCP has a narrow focus on weight loss	Well-being as the goal of the treatment, such as, for example, feeling fit and improving self-confidence and self-image
	HCP report that child and parents have high expectations on weight loss	Talking about their expectations of healthcare and behavior change process
		Take small steps and focus on other process-related results, creating successes and experiencing the benefits of a healthy lifestyle

**Table 4:** HCPs’ perceived barriers and facilitators related to the theme ‘Enhancing the patient-professional relationship’

**Sub-themes**	*Barriers*	*Facilitators*
**Investing in a relationship**	HCPs in secondary or tertiary care have less opportunity to build a relationship	Building trust and a strong relationship helps to support the process of finding out the underlying problems
	HCPs can have conflicts with parents over this sensitive subject	Having a personal connection with the child and parents
	HCPs report having contact mainly with parents	Involving both child and parents during the consult
	Building a relationship takes time	HCP respects and acknowledges families’ efforts
		Being equivalent partners: exploring together the situation (underlying problem, preferences, and options for change) and making a plan
		Creating a pleasant atmosphere, by, for example, using humor
		Creating a low threshold for contact, by, for example, doing consults with other HCPs together or making home visits
**Supportive attitude of the HCP**	HCP experiences the integrated care for children with obesity as complicated and intensive	HCP with affinity with obesity, behavior change and the psychosocial side of healthcare
	HCP experiences frustration, when unable to sufficiently help children with obesity	HCP has an open and curious attitude, displays empathy and withholds judgement
	HCP is pessimistic on perspectives for children with obesity	HCP perseveres, takes more initiative to engage in contact and stays in touch
	HCP experiences conversation about weight as difficult	Cultural sensitivity
		HCP feeling a sense of responsibility

**Table 5:** HCPs’ perceived barriers and facilitators related to the theme ‘Integrated care system’

**Sub-themes**	*Barriers*	*Facilitators*
**Network with dynamic roles and responsibilities**	Complexity of an integrated care network	The National model integrated care for childhood overweight and obesity
	Vague roles and responsibilities	
	Insufficient time for extra responsibilities within network	
	Malfunctioning of network	Having a coordinating professional
**Infrastructure for interdisciplinary collaboration**	Challenges associated with interdisciplinary communication	Building and maintaining close relationships within network
	Professionals in network regularly change	Regular multidisciplinary meetings
	Little feedback after referral	Warm transfer of information between professionals after referral
	Intra- and inter-disciplinary difference in approaches	A shared electronic patient file
**Time and insurance coverage as preconditions**	Insufficient time for providing adequate integrated care	Additional time for the coordinating professional
	Building and maintaining a network is time-consuming, while not being sufficiently reimbursed by insurance	HCP available in a flexible manner
		Additional time afforded by home visits are valuable for gathering information

### Exploring health, disease, and the illness experience

#### Barriers

HCPs perceived physical, mental, and social burdens associated with having obesity within many of the families. Furthermore, HCPs reported that the factors related to both the etiology and maintenance of obesity tend to be simplified by society, thus resulting in an underestimation of the complexity involved. In addition, problem awareness appeared to differ between families, asking for a tailored approach.

*HCP15, YHC-nurse: “Many children are suffering, they are really suffering; they are left out, they are being bullied, they are being chosen last at gym class or comments are made. […] Children tell their parents that they are being called a fatty or were not allowed to join […] So, what is this like for the parents when children come home with these stories? No parent likes that.”*

The HCPs reported that children often have an extensive history in healthcare and, as such, have seen many different professionals. Because of this, patients had repeatedly told the same painful story without receiving adequate care in return. When starting a healthcare trajectory, the HCPs perceived that both children and parents had high expectations regarding positive treatment outcomes, which can quickly result in experiences of failure. Subsequently, after having regularly experienced this sense of failure in trying to change their health behavior, the attitude of their patients appeared to change and their expectations were lowered. This process can have the same effect on HCPs themselves, insofar as when they are unable to adequately support their patients, they too can become discouraged.

*HCP01, pediatrician: “Well, what you see more often is that 14-year old’s have already experienced a longer period of being hurt or a longer period of being overweight. And often, they already have a wide range of negative experiences. Negative experiences with healthcare professionals or negative experiences with bullying or those kinds of things.”*

#### Facilitators

Facilitators that counteract the illness experience of obesity and prior healthcare experiences were found in the theme ‘Enhancing the patient-professional relationship’. When HCPs believed that children felt supported and acknowledged, then this appeared to help in terms of both building trust and preventing drop-out. The HCPs noted how they first sought to invest in building a relationship, to get a sense of the problems and priorities, before bringing up the topic of weight later in the conversation. In addition, paying explicit attention to managing expectations can help to reduce the sense of failure for children, parents, and HCPs, and loosen up the conversation.

*HCP08, YHC-nurse: “In the first conversation, I actually do not fully focus on the weight itself. I first want to get acquainted, build a relationship of trust and together look at what is going on, and what is needed, before I actually look at, what do we need to start up [treatment-wise]?”.*

The HCPs reported that measuring and weighing can be a negative experience for children, which might result in refusing to step on the scale. Alongside this, HCPs reported that when BMI is decreasing, then the weighing can also be a positive experience.

*HCP02, pediatrician: “She had not wanted to stand on the scale. So, I said to her: ‘Well, then we certainly will not do that. I mean, tell me what I can do for you?’ And that made her emotional. She said that ‘actually nobody had ever said that. Normally this was not an option.”*

### Understanding the whole person and its contexts

#### Barriers

The HCPs reported that, although common amongst other professionals, assessment, and treatment of solely nutrition and physical activity negatively affects the chances of successful health behavior change. However, their experience is that the complexity and lack of knowledge over how to identify and best treat underlying factors often serves as a barrier.

*HCP06, YHC-nurse: “You have a lot of children who went to see a dietician, who went to see a physiotherapist, who went to see whoever. But then they often have to make such big changes, they cannot sustain that. Well, and then they quit.”*

#### Facilitators

Beginning with an assessment of psychosocial and lifestyle factors in addition to biomedical factors helps to get the children, parents and professional acquainted with one another, in addition to aiding the HCP to work with a broad perspective that encompasses both the individual characteristics and environmental contexts of the child and parents.

*HCP09, YHC-nurse: “Horrible divorces, unemployment, family quarrels, poverty; there are many families where I have not yet talked about nutrition and health. I think all the other things need to go first, because otherwise there is no space in people’s head to also tackle this [the obesity]”.*

### Finding common ground

#### Barriers

HCPs reported the need to investigate what a family’s priorities are, which is often described as a difficult but nevertheless crucial step. During this investigation, HCPs seek to adopt a coaching role and place the child and parents in an expert role. However, some HCPs stated that not all families appear to be willing or able to do this. In addition, the HCPs reported that integrated care for children with obesity is a slow process, which requires building trust, letting the message sink in, and creating space for the family to take ownership. Whereas, some HCPs experienced that they found being patient challenging, and that their own work-ethic sometimes acted as a barrier, insofar as they sought to be fast, practical, and solution-focused.

*HCP02, pediatrician: “Then, you get a whole different conversation. […] Then, it turns out that the child is not happy. Then you can say, ‘I do not think we need to talk about your weight. That is not the main problem right now, what is now actually the most important thing for you?”.*

#### Facilitators

All HCPs tailored their approach to the characteristics of the family. When searching for underlying causes or hindering factors related to their lifestyle, the HCPs stressed the need to go beyond biomedical factors alone, to also include either individual psychological factors of the child and parents themselves or other factors in their social environment. Moreover, rather than narrowly focusing on weight loss, the HCPs noted that the scope should be broadened towards enhancing their well-being, while, simultaneously, paying explicit attention to the behavior change process by setting small goals, in order to build the confidence and energy needed for the child, parents and HCP to continue.

*HCP09, YHC-nurse: “You really need to stand next to people and together look at: where do you want to go and how long do you need me to walk next to you? But you lead the way.”*

### Enhancing the patient-professional relationship

#### Barriers

When trying to connect with children and parents, HCPs need the flexibility, creativity, and freedom needed to adjust and experiment within their practice. Some HCPs based their diagnostic and therapeutic steps partly on their gut-feeling, which necessitates a personal connection, while other HCPs reported that the lack of this personal connection can result in low motivation for the healthcare process, parental resistance towards the treatment, no-shows, and drop-outs.

*HCP11, YHC-nurse: “Connecting is searching”.*

The HCPs valued the involvement of both children and parents in the conversation, although attendance of only one or the other at appointments can sometimes be helpful, depending on their needs and the specific content to be discussed.

*HCP05, pediatrician: “This is about the approach and how people look at you. There is an enormous amount to gain. Because in the end, this is about: do I have a connection with this professional, will I dare to tell them what I actually do not dare to tell other people… That is what you want.”*

#### Facilitators

The HCPs reported that curiosity and the adoption of an inquiring attitude helps. An often-used word in the HCPs’ accounts was ‘together’: HCPs tried to place themselves on an equal level with the child and parents. When HCPs demonstrated their personal involvement with the families, they expressed feeling incredibly responsible for the well-being of these children. They also underscored the need to be determined and tenacious, as it is often a long and intensive process.

*HCP01, pediatrician: “They [the coordinating professionals] really take their role as a coordinating professional [seriously]. They sink their teeth into it and do not let go”.*

### Integrated care system

#### Barriers

The HCPs reported a difference between integrated care as described in the national model and the integrated care provided in practice. The HCPs perceived several collaborative challenges related to role dynamics, responsibilities, and available partners within the network. Also, infrastructural barriers were reported, such as, for example, insufficient intervention options and facilities for interdisciplinary communication. Moreover, HCPs experienced having insufficient time, because integrated childhood obesity care requires extensive energy and is time-consuming; in this respect, they expressed the need for additional funding for the time required to build and maintain relationships within their local integrated care network.

*HCP04, pediatrician: “Logistics take up so much time and energy! But it is one of the most important facets of the whole local integrated care and the treatment of obesity. So, these children do not fall through the cracks of the system. Those are the short lines [of communication within a network].”*

#### Facilitators

HCPs experienced working within an integrated care system as helping to address the complexity of childhood obesity, whereby a multidisciplinary team can bring a wide range of expertise as well as different ways of finding common ground with families.

## Discussion

This study explored the barriers and facilitators from the perspective of healthcare professionals (HCPs) providing integrated care for children with obesity. To the best of our knowledge, this is the first study that has explored this within integrated care for childhood obesity and provided a broad overview of its barriers and facilitators. The HCPs’ perceived barriers and facilitators can be clustered into fourteen sub-themes, which correspond to all four themes of the patient-centered care model, while the integrated care system makes up the fifth theme. Overall, the HCPs defined the etiology of obesity as complex, and experienced the integrated care as complicated. The main barriers were perceived within the sub-themes of illness and healthcare experiences, and sensitivity over talking about weight-related issues. The main facilitators were perceived within the sub-themes of conducting a biomedical, psychosocial and lifestyle assessment, tailoring the approach to families’ situation and investing in a family-professional relationship.

Our findings confirm the results from previous research conducted in other countries and other healthcare systems [32, 33]. These studies reported similar perspectives from HCPs, such as, for example, the importance of sensitive communication, the influence of obesity stigma and inadequate organizational resources. Our findings add insight into both the perceived complexity of the etiology of childhood obesity and the complicated nature of integrated childhood obesity care [34]. Complexity is a well-known subject within obesity research, of which the Foresight Obesity System map is perhaps the most famous example: this map illustrates the etiology and maintenance of obesity by incorporating 108 factors and more than 300 interdependent relationships [35], ranging from individual biological to psychosocial factors, and being influenced by socio-economic, cultural and environmental conditions [36]. As the obesogenic environment also plays an important part in health behavior, integrated care needs to form part of a whole systems approach that incorporates the prevention of obesity by creating a healthy environment for children in general, and integrated care for the individual child with obesity and their parents [37–39]. Although our study shows that knowledge over the complexity of influencing factors in relation to obesity does not necessarily make the treatment experience any less complicated [40]. Within the present study, working within an integrated care system was remarkably regarded as both a facilitator and a barrier, depending on the level of implementation. The facilitators that HCPs perceived were bringing together different expertise and thereby also different ways of establishing common ground with families. However, the perceived barriers identified by HCPs pertain to the challenges associated with interdisciplinary collaboration within the integrated care network, in addition to how insufficient time and lack of health insurance coverage hinder the adequate implementation of integrated care.

Obesity carries a social stigma [41, 42], which appeared to be an underlying barrier for HCPs that impacted, both explicitly and implicitly, upon all themes. Although obesity stigma in itself was not often literally mentioned, HCPs indirectly acknowledged its presence. At the individual patient level, HCPs reported that children spoke of being regularly bullied and experiencing obesity-related physical, mental, and social limitations. At the social level, the HCPs reported that children and parents often expressed how they felt like they were being looked at, or criticized based on their weight and health behaviors in many contexts. At the healthcare level, HCPs frequently reported the negative prior healthcare experience that children and their parents have had. This indicates that HCPs themselves can also be the source of obesity stigma, due, in part, to the influence of public obesity stigma or personal prejudices [43, 44]. Within the present study, the HCPs indicated that they were aware of the importance of adopting a non-judgmental attitude, but yet reported that other professionals make judgements regarding people with obesity and obesity care. These attitudes can influence the person-perceptions, judgment, interpersonal behavior, and decision-making of HCPs [33, 45]. Moreover, experiences with perceived obesity stigma, both outside and within the healthcare context, could result in anticipated and internalized stigma [46] and appear to influence children and parents’ behavior, insofar as the professionals noted that families often seemed to be on guard or cautious. Therefore, paying attention to obesity stigma is of paramount importance, inasmuch as it can result in negative physical and psychological consequences, receiving less adequate care and creating a negative feedback loop, which, in turn, influences health-related behaviors, causes weight gain or prevents weight loss [33, 47].

### Patient-centered childhood obesity care in clinical practice

Our findings have implications for clinical practice. The results of this study support the use of the patient-centered care model to structure a tailored approach within integrated care. When a HCP uses the identified themes when organizing and offering integrated care, a broad perspective on individual characteristics and environmental contexts will be adopted and extra attention will be given to investment in the relationship, with respect to the sensitivity and complexity of the disease obesity.

When exploring the health, disease and illness experiences of children (Fig. 1, theme 1), in the patient-centered care model a distinction is drawn between the pathophysiologic processes of the disease and the personal subjective experience of the illness [19, 48]. In the case of obesity, the disease is determined by a multifactorial disbalance between energy intake and expenditure, which interferes with a healthy metabolism. While the illness experience pertains to someone’s personal experience of the physical, emotional, and mental burdens of having obesity, as perceived by the professionals in this study. As a healthcare professional, acknowledging this illness experience of obesity along with its possible influencing individual characteristics and environmental contexts can form the basis of finding a common ground (Fig. 1, theme 3) between children, parents, and professionals regarding how to specifically tailor the healthcare approach. When attempting to find this common ground between children, parents, and professionals, HCPs support and empower the child and parents to set their own behavior change goals, based on their own personal interests and values. Encouraging self-management outside of the healthcare context can be achieved by facilitating ownership and following the priorities of the family [49]. In so doing, a healthcare professional can create an autonomy supportive healthcare climate, one which motivates the onset and long-term sustainment of self-determined health behaviors [50, 51]. When trying to understand the whole person and its contexts (Fig. 1, theme 2), in clinical practice it is helpful to conduct an assessment of psychosocial and lifestyle factors [52]. This serves to generate awareness amongst children, parents, and professionals regarding the complexity of factors related to the etiology and maintenance of obesity, which, in turn, can enhance empathy and understanding of the disease. When enhancing the family-professional relationship (Fig. 1, theme 4), a professional can seek to mitigate the sensitivity related to talking about weight-related issues by taking the time to build a relationship and using emphatic communication with respectful weight-related terminology [53, 54]. For all these themess, professionals must be educated on the illness experience of obesity, knowledge, and skills if they are to be a responsive, coaching partner in the family-professional relationship [19]. To reduce obesity stigma, HCPs’ educational programs should address the etiology and biology of obesity as well as the environmental determinants of obesity-related behaviors, stereotypes and fears about obesity, and other drivers of stigma [46].

However, practicing patient-centered childhood obesity care within single healthcare settings is insufficient. Rather, it must take place within the integrated care system (Fig. 1, theme 5) and be adequately supported, both financially and in terms of time. Of course, this call for additional finance and time is far from new [55]; however, what our study illustrates once again is that the extensive process of implementing and practicing patient-centered care requires sufficient time. Although adopting integrated care is viewed as facilitative, when this approach is not wholly implemented and the care is not reimbursed, it can also serve as a barrier by virtue of adding to the complicated nature that professionals already experience, when HCPs are unable to meet the integrated care networks’ demands.

### Strengths & limitations

One limitation of the study is that data saturation was not always reached. This may have been the result of both the wide range of topics that were covered and the heterogeneity of the participants’ experiences, which, alternatively, can also be seen as a strength. Second, this study included a specific group of relatively experienced HCPs. In that sense, we do not know whether this patient-centered care structure fits both professionals with and without this specific expertise, insofar as it is likely that HCPs with less experience may perceive more theoretical barriers, while being unaware of practical barriers. Third, this study explored HCPs’ perspectives of children’s experiences, rather than gaining insight into patients’ own experiences [56, 57]. Therefore, further participatory research with children is urgently required [58]. The strengths of this study are that, at least to the best of our knowledge, this is the first study using the patient-centered care model to analyze the perspectives of HCPs providing integrated care for children with obesity [19]. Last, given that interdisciplinary collaborations are essential within integrated care, a further strength of our study is that we included the views of HCPs from different professions, who are working in a variety of smaller and larger municipalities. In addition to the multidisciplinary group of authors, the complexity of integrated care was studied by a team with complementing expertise. The researcher that interviewed participants is an psychologist with expertise on health promotion and behavior change. The researchteam included further experts on obesity care, pediatric care, endocrinology, health sciences, clinical psychology, nutritional science and public health policy.

## Conclusions

In conclusion, healthcare professionals (HCPs) providing integrated care for children with obesity, define the etiology of obesity as complex and experience integrated care as complicated. Many barriers and facilitators were perceived with respect to both the four themes of the patient-centered care model and the fifht theme of the integrated care system. The sensitivity of weight-related issues implicitly affected both the illness and healthcare experience, while explicitly impacting on conversations about childhood obesity. The results of this study support the use of the patient centered care model to structure a tailored approach within integrated care. This approach supports HCPs in adopting a broad perspective on individual characteristics and environmental contexts and investing in a relationship, with respect to the sensitivity and complexity of the disease obesity.

## Supplementary Information

Supplementary Material 1.

## Data Availability

All data generated or analyzed for the purposes of this study are included in this published article as well as the supplementary information files. The data analyzed for the current study are available upon reasonable request from the corresponding author (emma.vandeneynde@jogg.nl).

## Abbreviations

HCP: Healthcare professional
YHC: Youth healthcare

## Data availability

The dataset supporting the conclusions of the current study is available for researchers who meet the criteria for access to data upon request which can be applied at the Data Access Committee of C-support.
