# Supplementary material for: Healthcare professionals’ perspectives on the barriers and facilitators of integrated childhood obesity care
Source: BMC Health Serv Res. 2024 Sep 27;24:1133. doi: 10.1186/s12913-024-11532-9 (PMC11428901; doi:10.1186/s12913-024-11532-9)
Supplement: Supplementary file 1 — Supplementary Material 1. [file 12913_2024_11532_MOESM1_ESM.pdf]

Supplementary Material 1. Interview protocol

van den Eynde et al, (2024) HCPs on barriers facilitators childhood obesity care - BMCHSR

Opening

Informed consent information

With your permission the interviews will be audio-recorded. The recording will be treated confidentially. The audio will be transcribed and anonymized so the transcript is not traceable to your personal data. If you wish to end the interview or take a break, you are free to do so at any time. If you agree, please sign the informed consent form. Do you have any questions before we begin?

Introduction interviewer

The interviewer studied Psychology of Health Behavior with a special focus on (childhood) obesity, and has experience in childhood obesity intervention development, coordination and implementation, and obesity care research. Now, she works as a PhD candidate.

Purpose of the research

Purpose of research consortium

The research consortium is focused on stimulating a healthy lifestyle (nutrition, physical activity and sedentary and sleeping behavior) in children aged 10-14, with a lower socio-economic position, from different ethnical backgrounds. The goal of the program is to change the environment in a way that healthy behavior is encouraged and unhealthy behavior is discouraged. Within the consortium, we work together with children themselves, but also with other relevant stakeholders (including parents, schools, supermarkets and professionals)

Purpose of subproject

The purpose of this particular research project is to explore the needs and possibilities around support, care and empowerment that leads to a tailored approach for sustainable lifestyle change. This is explored together with children with obesity, their parents and healthcare professionals. The goal is to unite the needs of children with obesity and their parents and the current care and support provided by healthcare professionals.

Purpose of these interviews

The purpose of this interview is to (1) discuss the hindering and helping factors, needs and possibilities that healthcare professionals observe in children with obesity and their parents in achieving a healthier lifestyle, and (2) the hindering and helping factors, needs and possibilities that healthcare professionals experience themselves in supporting and empowering children with obesity and their parents in achieving a healthier lifestyle. In addition, we will discuss the difference between 10- and 14-year-old children.

Questions

Opening question

- To start, could you tell a bit about the work you do with children with obesity?
    - What is your role within the integrated care approach?
    - How often do you see children with obesity in your work?

Topic 1: Child and family

Barriers

- In your experience, what are factors that make it difficult for children with obesity and their parents to make healthy choices?
    - Difference between 10 and 14?
    - How do children with obesity and their parents deal with these factors?

Facilitators

- From your point of view, what are important factors that help children with obesity and their parents to make healthy choices?
    - Difference between 10 and 14?

Needs

- What do they need according to you, to make healthy choices and overcome these barriers?

Possibilities

- Where do you see possibilities for change?

Topic 2: Healthcare professionals

Barriers

- Which factors make it more difficult for you to support and empower children with obesity and their parents?
    - Difference between 10 and 14?

Facilitators

- Which factors make it easier for you to support and empower children with obesity and their parents?
    - Difference between 10 and 14?

Needs

- What do you need to provide the necessary support, care and empowerment for these children?
- What do you need to overcome the barriers you mentioned previously?
- What do you need to create more facilitators?

In case the role of the coordinating professional has not been discussed yet:

- What does a coordinating professional need to:
    - Provide the necessary support, care and empowerment?
    - Overcome the barriers?
    - Create more facilitators?
- According to you, who should have the role of coordinating professional in the care for children with obesity aged 10 to 14?

Possibilities

- Where do you see possibilities for change?

Closing question

- Are there any subjects that we haven't discussed that you would like to talk about?

Personal information

- For how long have you been working in the field of childhood obesity?
- Age?

Probes

Probing questions are based on the socio-ecological model (REF: Bronfenbrenner, 1979), including three different levels: (1) intra/inter personal, (2) organization/institution, (3) society.

Probing questions in relation to needs are based on the Self-Determination Theory including three basic psychological needs: competence, relatedness and autonomy (REF: Ryan & Deci, 2000)
